# High-Risk Leukemia: Past, Present, and Future Role of NK Cells

**DOI:** 10.1155/2018/1586905

**Published:** 2018-04-15

**Authors:** Melissa Mavers, Alice Bertaina

**Affiliations:** ^1^Division of Stem Cell Transplantation and Regenerative Medicine, Department of Pediatrics, School of Medicine, Stanford University, Stanford, CA, USA; ^2^Bass Center for Childhood Cancer and Blood Diseases, Stanford Children's Health, Palo Alto, CA, USA; ^3^Stem Cell Transplant Unit, Department of Hematology and Oncology, Bambino Gesu' Children's Hospital, Rome, Italy

## Abstract

Natural killer (NK) cells are a population of cytotoxic innate lymphocytes that evolved prior to their adaptive counterparts and constitute one of the first lines of defense against infected/mutated cells. Several studies have shown that in patients with acute leukemia given haploidentical hematopoietic stem cell transplantation, donor-derived NK cells play a key role in the eradication of cancer cells. The antileukemic effect is mostly related to the presence of “alloreactive” NK cells, that is, mature KIR+ NK cells that express inhibitory KIR mismatched with HLA class I (KIR-L) of the patient. A genotypic analysis detecting KIR B haplotype and the relative B content is an additional donor selection criterion. These data provided the rationale for implementing phase I/II clinical trials of adoptive infusion of either selected or ex vivo-activated NK cells, often from an HLA-mismatched donor. In this review, we provide a historical perspective on the role played by NK cells in patients with acute leukemia, focusing also on the various approaches to adoptive NK cell therapy and the unresolved issues therein. In addition, we outline new methods to enhance NK activity, including anti-KIR monoclonal antibody, bi-/trispecific antibodies linking NK cells to cytokines and/or target antigens, and CAR-engineered NK cells.

## 1. Introduction

A substantial body of evidence has emerged delineating the role of natural killer (NK) cells in the immunosurveillance of/immune response to leukemia as well as its therapeutic treatment. The role for NK cells in this setting is a consequence of their inherent biology. NK cells are a hallmark component of the innate immune system and possess both activating and inhibitory receptors recognizing molecular structures on cell surfaces [1]. In particular, inhibitory receptors which recognize HLA class I molecules play an important role in their function. These inhibitory killer cell immunoglobulin-like receptors (KIR) permit NK cells to recognize “self” and provide inhibitory signals to preclude killing of the target cell [2]. When cognate HLA class I molecules are absent, and no inhibitory signal is provided, signals from activating receptors are unopposed and can lead to NK cell activation and target cell killing [3]. Thus, in the setting of anticancer immunity, those target tumor cells which have downregulated HLA class I may be a prime target of NK-mediated immunity [4]. Further, in a hematopoietic stem cell transplantation (HSCT) setting, donor NK cell inhibitory receptors mismatched for cognate HLA class I ligand play an important role in the graft-versus-leukemia (GvL) effect [5]. These cells may be uniquely poised to enhance GvL without eliciting graft-versus-host disease (GvHD) because healthy nonhematopoietic tissues lack activating receptor ligands present on tumor cells [6]. Therefore, exploiting the properties of these cells may permit an enhancement of cancer immunity. Herein, we describe the key role played by NK cells in the setting of haploidentical (haplo) HSCT as protection against leukemia recurrence, review the adoptive transfer of NK cells for leukemia immunotherapy with or without HSCT, and enumerate the novel approaches being investigated to enhance NK activity.

## 2. The Role of NK Cells in Haploidentical HSCT to Cure High-Risk Leukemia: The Importance of Donor Selection

**Figure 1:**
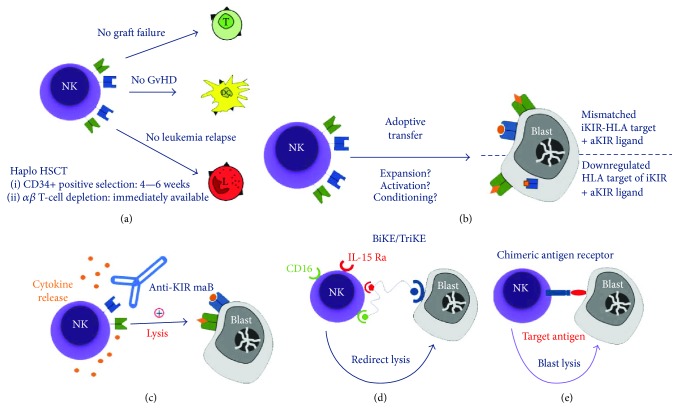
Mechanisms of natural killer (NK) cell-mediated antileukemic effect. Natural killer cells play a significant role in immunological defense against leukemia. (a) Following haploidentical hematopoietic stem cell transplantation, donor NK cells facilitate engraftment by eliminating recipient T cells, fail to elicit graft-versus-host disease (GvHD) by eliminating recipient dendritic cells, and suppress leukemia relapse through a direct cytotoxic effect on leukemic blasts, particularly in the setting of inhibitory killer immunoglobulin-like receptor (KIR) mismatch which leads to a failure of inhibitory signaling in the NK cell. (b) Adoptive transfer of NK cells leads to leukemic blast killing through either inhibitory KIR mismatch or downregulation of inhibitor KIR ligands (MHC) by the leukemic blast, as well as the provision of ligands for activating receptors present on the blast surface. Questions remain as to the best approach to NK cell expansion and activation as well as patient conditioning for adoptive transfer. Several methods to boost NK cell activation are under investigation, including (c) anti-inhibitor KIR antibody, (d) bispecific and trispecific killer engagers (BiKEs and TriKES) linking NK cells to target cells and providing activating signals, and (e) the generation of NK cells possessing a chimeric antigen receptor to target leukemia antigen. Green = activating KIR (on NK cells) or KIR ligand (on target cells); blue = inhibitory KIR (on NK cells) or KIR ligand (on target cells).

Hematopoietic stem cell transplantation from both matched related and unrelated donor has been widely employed for treating patients with acute leukemia, as well as many different severe nonmalignant disorders [7]. However, only 25% of the patients who need an allograft have an HLA-identical sibling, and a suitable HLA-matched unrelated donor can be identified for less than two-thirds of the remaining patients [8]. For those patients lacking an HLA-matched donor, alternative sources of hematopoietic stem cells (HSC) such as unrelated umbilical cord blood (UCB) and HLA-haploidentical relatives are being increasingly employed [8–10]. Indeed, UCB with up to 2 antigen mismatches can be used due to their reduced capacity to mediate GvHD. In addition, the majority of patients have a relative with one identical HLA haplotype and the other fully mismatched, namely, haploidentical (haplo), who can promptly serve as a donor of HSC [11, 12]. However, in the haplo setting, the significant immunogenic disparity between donor and recipient can lead to increased GvHD induced by mature donor T cells in the graft [13, 14]. Strategies to prevent GvHD after haplo HSCT based on either pharmacologic immunosuppression or T cell depletion of the graft have been developed. A breakthrough in the history of haplo HSCT was the demonstration that an efficient T cell depletion of the graft is able to prevent both acute and chronic GvHD [15, 16]. However, the absence of mature T cells from the graft jeopardizes immune recovery and antileukemia immune surveillance. This observation was only partly confirmed by clinical results, because early studies in adult acute myeloid leukemia (AML) showed that, in this setting, the GvL effect was due to the *in vivo* maturation of NK cells derived from donor HSC [16] (Figure 1). Remarkably, in haplo HSCT, an efficient GvL effect was detected only in patients receiving a transplant from a donor who presented alloreactive NK cells against recipient cells [17]. This happens, for example, in the presence of a KIR-HLA-I (KIR-L) mismatch in the donor-versus-recipient direction. Thus, in donor/patient pairs with KIR-HLA-I mismatch, the leukemia-free survival was significantly higher (60%) than in the absence of such mismatch (<5%).

Given the critical role of alloreactive NK cells in the GvL effect for patients undergoing T cell-depleted haplo HSCT, quantification and phenotypic analysis of NK cells in potential donors would enhance donor selection. Analysis of NK cytolytic activity could further inform anticipated GvL efficacy. [18]. Quantification of alloreactive NK cells can be accomplished through flow cytometry using appropriate combination of anti-KIR monoclonal antibodies (mAbs). [19, 20]. Pilot studies pointed at donor NK cell analysis of inhibitory KIR specific for HLA-I alleles lacking in patient cells. More recent reports highlighted the importance of some activating KIR. In this context, the recent availability of mAbs allowing discrimination between inhibitory and activating KIR resulted in an even more accurate definition of the alloreactive NK cell subset. As a matter of fact, the identification of activating KIR proved to be important for the clinical outcome when donors expressed KIR2DS1 and the patient expressed HLA-C2 alleles, representing the ligands of such activating KIR [18, 21–23].

In 2008, Stern and colleagues retrospectively analyzed both children and young adults suggesting an advantage in using as a donor the mother in comparison with the father [24]. This effect was independent of NK alloreactivity and may be due to peripartum exposure of the maternal immune system to fetal antigens derived from the paternal HLA haplotype, leading to development of memory T cell immunity [25].

### 2.1. NK Cell Alloreactivity and beyond

Over 20 years ago, Moretta et al. first described NK cell alloreactivity, demonstrating the *in vitro* association between defined NK cell subsets and the lysis of allogeneic lymphoblasts [26]. As noted above, alloreactive donor NK cells expressing inhibitory KIR mismatched for recipient HLA class I KIR ligands and possessing critical activating KIR positively impact the outcome of transplantation. Indeed, pediatric studies have also highlighted the importance of employing a donor with NK alloreactivity in haplo HSCT. A study was performed by The Acute Leukemia and Pediatric Diseases Working Parties of the European Blood and Marrow Transplantation (EBMT) Group which analyzed the outcome of a large cohort of children with acute lymphoblastic leukemia (ALL) given a CD34^+^ positively selected haplo HSCT [27]. This study showed a 5-year leukemia-free survival (LFS) of about 30% for children transplanted in complete remission. Furthermore, this study demonstrated that the HSC dose infused in haplo HSCT is crucial, as patients receiving a dose of CD34^+^ cells greater than 12 × 10^6^ progenitors/kg have a better clinical outcome. In 66 patients, sufficient information on HLA-C, -Bw4, and -Bw6 typing was available to classify patients into KIR ligand matching between donors and recipients. KIR ligand was matched with the donor in the GvHD direction in 41 patients and mismatched in 25. In this cohort, KIR mismatch in the donor/recipient pairs was not associated with a lower relapse incidence. More recently, Locatelli's group analyzed the outcome of 72 pediatric patients given a CD34^+^ positively selected haplo HSCT for the treatment of acute leukemia (ALL 42 cases and AML 21 cases) or myelodysplastic syndrome (MDS, 9 cases) transplanted at the Pediatric Hematology and Oncology, Policlinico San Matteo, Pavia. As presented during the 2011 EBMT Annual Meeting by Bernardo et al., the 5-year estimate of LFS for the whole cohort was 55% (61% in ALL, 33% in AML, and 78% in MDS patients, *p* < 0.01). Remarkably, in children with acute leukemia who received an allograft from a NK alloreactive donor, LFS was significantly better as compared to children who received an allograft from a non-NK alloreactive donor (63% and 35%, respectively, *p* = 0.05).

NK cell alloreactivity occurs only in around 50% of donor/patient pairs. In an attempt to compensate, at least in part, for the lack of alloreactivity, additional selection criteria have been added. Indeed, a wide range of receptors mediating either inhibitory or activating signals is involved in NK cell function [28]. Among these, the KIRs are of particular importance. Activating forms of KIRs have been identified and cloned, but only KIR2DS1 and KIR2DS4 that have the specificity for HLA class 1 molecules have been unequivocally documented [29]. Two basic KIR haplotypes can be found in humans: the group A haplotype, which has a fixed number of gene-encoding inhibitory receptors (with the exception of the activating receptor KIR2DS4), and the group B haplotypes, which have variable gene content and 1 or more of the B-specific genes: KIR2DS1, KIR2DS2, KIR2DS3, KIR2DS5, KIR2DL2, and KIR2DL5 [6, 30]. Among haplotype B individuals, a KIR B-content score can further be established on the basis of the number of centromeric and telomeric KIR B haplotype motifs.

Cooley et al. recently reported a reduced relapse risk in adults with AML, but not in those with ALL, given allogeneic HSCT from unrelated KIR haplotype B donors [31]. In 2014, Oevermann and colleagues analyzed the influence of donor KIR gene haplotypes on the risk for relapse and the probability of EFS in children with ALL who received haplo HSCT with ex vivo T cell-depleted peripheral blood stem cells [32]. Of the 85 donors who underwent KIR genotyping, 63 were of KIR haplotype B. Children transplanted from these donors had significantly better EFS than those receiving a graft from a KIR haplotype A donor (50.6% versus 29.5%, respectively; *p* = 0.033). Further, risk of relapse was significantly reduced in patients receiving a transplant from a donor with a KIR B-content score greater than 0 (log-rank test for trend, *p* = 0.026). Nowadays, this additional selection criterion is routinely applied by several groups for optimizing donor selection.

### 2.2. The Impact of NK Cell Reconstitution after HSCT

Moving to the role of NK cells after HSCT, it is now well known that donor-derived alloreactive NK cells are generated in patients and can persist more than 4 years after transplantation. In 2009, Pende and colleagues analyzed 21 children with leukemia receiving a haplo HSCT [21]. All donors were selected on the basis of the expression of a KIR for which the cognate ligand (HLA allele) was missing in the patient. In this pediatric cohort, most patients who displayed a posttransplant high proportion of donor-derived alloreactive NK cells remained leukemia-free. Notably, a correlation was found between the size of the alloreactive NK subset and the clinical outcome.

In the adult setting, it has been reported that engrafted stem cells regenerated the same repertoire as was present in the donor, including donor-versus-recipient alloreactive NK cells for up to 1 year [33]. In an updated analysis, 112 high-risk AML patients received haploidentical transplants from NK alloreactive (*n* = 51) or non-NK alloreactive donors (*n* = 61) [5]. Transplantation from NK alloreactive donors was associated with a significantly lower relapse rate in patients transplanted in complete remission (3% versus 47%; *p* < 0.003), better EFS in patients transplanted not only in remission (67% versus 18%, *p* = 0.02) but also in relapse (34% versus 6%, *p* = 0.04), and overall reduction of the risk of relapse or death (*p* < 0.001). The 67% probability of EFS for AML patients transplanted in remission from NK alloreactive donors is in the range of the best survival rates achieved after transplantation from unrelated donors and UBC. The *in vitro* resistance of common phenotype ALL to alloreactive NK killing was paralleled by lack of antileukemia effect in adult patients. However, in ALL in children, transplantation from NK alloreactive donors was reported to decrease the risk of relapse [24].

Indeed, the NK cell-mediated lysis of tumor cells involves several different receptors, according to the type of malignancy. It has been described, for example, that the recognition and lysis of AML blasts occur mainly through the NCR and DNAM-1 receptors [34]. On the contrary, the pathways of NK cell/ALL blast interaction remain to be better clarified. In order to address this question, Torelli and colleagues investigated the pathways of ALL blast recognition by NK cells. They evaluated whether a correlation existed between expression of various NK cell-activating receptor ligands by leukemic blasts and their ability to be recognized and killed by NK cells [35]. A differential expression of known NK cell-activating receptor ligands between pediatric and adult ALL blasts was demonstrated. Increased expression of Nec-2, ULBP1, and ULBP3 was noted in blasts from pediatric patients relative to blasts from adults. This increased expression of some NKG2D and DNAM-1 ligands in pediatric samples may provide an underlying mechanism for the observation that use of an NK alloreactive donor reduces ALL recurrence in pediatric but not in adult patients [12]. Remarkably, the differential expression of these ligands between adults and children is limited to B-lineage ALL, while no differences were observed within T-ALL [35].

Thus, while the absence of the T cell-mediated GvL effect was for many years considered to render the recipients of a T cell-depleted allograft more susceptible to leukemia relapse, it is now evident that an efficient GvL effect can be mediated by donor-derived alloreactive NK cells without an association with GvHD (Figure 1(a)). Indeed, NK cells kill leukemia blasts but spare normal tissues with the important exception of hematopoietic cells [6]. It is conceivable that this selective effect is due to either the lack of or low expression of ligands engaged by the activating NK receptors in normal, nonhematopoietic cells. This would explain why alloreactive NK cells kill patient dendritic and T cells, thus preventing GvHD and graft rejection, respectively.

### 2.3. New Strategies of Graft Manipulation in Haploidentical HSCT: A New Source of NK Cells

In the haplo HSCT setting, after the infusion of positively selected CD34^+^ cells, the first appearance of endogenous KIR+ alloreactive NK cells may require at least 1 month delaying their potential antileukemic effect [36]. It is evident that, in cases of high residual tumor burden and/or of rapidly proliferating leukemia blasts, this may result in leukemic relapses. In the attempt to overcome this risk, a very promising approach has been recently developed. This new technique of graft manipulation is based on the association of a negative depletion of T lymphocytes carrying the *αβ* chains of the T cell receptor (TCR) and of a CD19^+^ B cell depletion [36]. Indeed, T lymphocytes carrying the *αβ* chains of TCR are the only subset responsible for the occurrence of GvHD [37, 38]. Thus, their physical elimination from the graft translates into the prevention of this life-threatening immune-mediated complication. This novel approach allows the transfer to the recipient not only of a “megadose” of CD34^+^ cells but also of mature donor NK cells and *γδ* T cells. Both these lymphocyte subsets are immediately available and can display a protective effect against leukemia regrowth and severe infections, especially in the very early phase after haplo HSCT [39, 40] (Figure 1(a)). The first systematic study on this approach was recently carried out by the group of Locatelli at the Bambino Gesu' Children's Hospital in Rome [41]. They reported the outcome of 80 children given an *αβ* T cell- and CD19^+^ B cell-depleted haplo HSCT (*αβ*/CD19^+^ haplo HSCT) and compared their results with those of 41 and 51 children transplanted in the same period with an HLA-identical sibling donor or a 10/10 allelic-matched unrelated donor, respectively. In the *αβ*/CD19^+^ haplo HSCT cohort, the cumulative incidence of grade 1-2 acute GvHD was 30% (all skin only), while no patient developed grade 3-4 acute GvHD or extensive chronic GvHD. The cumulative incidence of nonrelapse mortality was 5% (with 4 deaths due to idiopathic pneumonitis in 2 cases, disseminated adenovirus infection after graft failure, and cardiac insufficiency), with a 24% cumulative incidence of relapse. With a median follow-up of 46 months, the 5-year probability of chronic GvHD-free, relapse-free survival (GRFS) for *αβ*/CD19^+^ haplo HSCT was 71%. In comparison with HLA-identical sibling and 10/10 allelic-matched unrelated donor transplants, *αβ*/CD19^+^ haplo HSCT had a lower incidence of grade 3-4 acute GvHD, of visceral GvHD, and of chronic GvHD. The 5-year probability of LFS and GRFS did not differ between *αβ*/CD19^+^ haplo HSCT recipients and the other 2 cohorts of patients, indicating that haplo HSCT after *αβ* T and B cell depletion represents a competitive alternative for children with acute leukemia in need of urgent allograft [41]. Other experimental demonstration of the efficacy of this graft engineering technique was reported by Maschan's group, in which the outcome of children with high-risk AML, who received transplantation from unrelated and *αβ*/CD19^+^ haploidentical donors, was analyzed in 28 patients [42]. Pharmacologic immune suppression with tacrolimus and methotrexate was employed post-HSCT. Notably, recipients of haploidentical grafts more commonly developed isolated skin GvHD, whereas gastrointestinal involvement was more frequent in unrelated HSCT. In the 13 haplo HSCT recipients, the cumulative incidence of relapse at 2 years was 40% (95% CI: 20–80), whereas the LFS probability was 59% (95% CI: 31–87). Moreover, Lang et al. recently published a retrospective analysis of immune recovery in a cohort of 41 pediatric patients affected by either acute leukemia, myelodysplastic syndrome, or nonmalignant diseases (*n* = 55), who received *αβ* T and B cell-depleted allografts from a haploidentical relative after reduced-toxicity regimens [43]. Severe acute GvHD occurred in 15% of patients, with a median follow-up of 1.6 years; 21 of the 41 patients were alive and relapse was the major cause of death.

## 3. Adoptive Immunotherapy with NK Cells

Given the important role that NK cells play in cancer immunity, it is no wonder that many groups have explored adoptive immunotherapy of NK cells to boost tumor immunity (Figure 1(b)). The majority of these studies to date have focused on the safety and feasibility of NK cell adoptive immunotherapy with a variety of NK manipulations and in a number of clinical settings, including low-/medium-risk patients and high-risk patients, with or without HSCT, and pediatric versus adult patients. In addition, most groups have selected donor/patient pairs based on inhibitory KIR mismatching or in a subgroup analysis have found that inhibitory KIR mismatching led to the best outcomes. These studies are summarized below.

### 3.1. Pediatric and Young Adult Studies

Among the first to explore NK adoptive immunotherapy were the groups at Frankfurt University and Basel University Hospital, focusing on pediatric and young adult patients. A 2004 pilot study reported 3 children (2 with ALL and 1 with AML) who underwent NK adoptive immunotherapy following CD34^+^ selected haplo HSCT [44]. Each patient received 1–3 infusions of KIR-mismatched (in the GvL direction) haplo NK cells ranging from 3.3 × 10^6^/kg to 29.5 × 10^6^/kg per dose starting on day +1 or +2 and every 4–6 weeks thereafter. Nonmobilized apheresis products had undergone two rounds of magnetic-bead-based CD3^+^ depletion followed by CD56^+^ positive selection and were cultured in the presence of IL-2 for 2 weeks (median 5-fold expansion), with all CD3^+^ doses under 2 × 10^4^/kg. Patients received IL-2 *in vivo* following adoptive transfer. The AML patient died of relapse (day +80), while the two ALL patients died of transplant-related complications. Aside from one patient developing a mild exanthema, possibly due to parvovirus infection versus mild skin GvHD, no other GvHD was noted and no infusion-related adverse events (AE) occurred. At the same time, another series of 5 pediatric/young adult patients (4 AML and 1 chronic myeloid leukemia (CML)) focused on use of NK adoptive transfer to consolidate engraftment following haplo HSCT (CD34^+^ selected and CD4^+^ and CD8^+^ depleted), with primary endpoints of safety and feasibility [45]. Patients were enrolled for mixed chimerism (3), graft failure (1), and early relapse (1). Apheresis products from the stem cell donor were CD3^+^ depleted and CD56^+^ selected, and most were split into 2 infusions 2 months apart, with a median of 9.3 × 10^6^/kg NK infused (median 0.22 × 10^5^/kg CD3^+^, max 0.55 × 10^5^/kg). No *in vitro* or *in vivo* cytokine exposure was incorporated into the study. Three of the five donors were predicted to have NK alloreactivity based on KIR/KIR ligand mismatch. No infusion-related AE, GvHD, or infections were noted. Whole-blood donor chimerism increased in 2 patients (one of whom also received a stem cell boost), stabilized in 1 patient, and decreased in 2 patients (one with early relapse). At a median of 12 months of follow-up, 4 of the 5 remained alive and in remission. These two pilot studies demonstrated safety and feasibility and led to a multicenter phase II trial [46]. Sixteen high-risk patients were enrolled (8 AML, 5 ALL, 2 Hodgkin's lymphoma (HL), and 1 sarcoma), and active disease was present in 44% (2 had relapsed after prior allogeneic HSCT). NK infusions (derived from the original donor and CD3^+^ depleted/CD56^+^ selected, with no *in vitro*/*in vivo* cytokine exposure) were performed on post-haplo HSCT days +3, +40, and +100 at one center and on days +40 and +100 at the other center. KIR mismatch was only present in 11/16 donor/recipient pairs. Median NK dose was 12.1 × 10^6^/kg with median T cell dose 0.03 × 10^5^/kg (max 0.72 × 10^5^/kg). No infusion reactions were noted, though 4 patients developed grade II–IV acute GvHD (3 were fatal). Notably, all patients who developed GvHD received a cumulative T cell dose (from HSCT and NK infusion) of >0.5 × 10^5^/kg, while none of the patients with <0.5 × 10^5^/kg developed significant GvHD. With a median follow-up of 5.8 years, 4/8 AML and 1/4 ALL patients experienced relapse. Twelve of the 16 patients died, with causes of death including relapse (5), GvHD (3), graft failure (3), and transplant toxicity (1). The presence or absence of KIR mismatch appeared to have no effect on risk of graft failure or relapse. In addition, NK infusion had no apparent effect on rates of graft failure or relapse compared with historical controls who underwent haplo HSCT without NK infusion.

The group at St. Jude also performed a pilot study in which 10 patients with AML in first complete remission (CR) not receiving HSCT (4 low risk, 6 intermediate risk) underwent haplo NK cell adoptive transfer after conditioning with cyclophosphamide and fludarabine [47]. Apheresis products (CD3^+^ depleted/CD56^+^ positively selected) were immediately infused, followed by *in vivo* IL-2 treatment. Patients received a median of 29 × 10^6^/kg NK cells, with 9 of 10 donor/recipient pairs mismatched for inhibitory KIRs. There was an acceptable rate of nonhematologic toxicity (1 viral infection, 1 febrile neutropenia, and 1 IL-2 injection site reaction) with no GvHD or infusion reactions. All had transient donor NK engraftment (median 10 days) with median donor chimerism peak at 7%. Expansion of donor NK cells was noted in all KIR-mismatched settings, and all patients remained in remission with median follow-up of 964 days. Subsequently, this group also evaluated this approach in the setting of relapsed/refractory leukemia (8 ALL, 6 AML, including 9 with high blast burden) or relapsed disease following allogeneic HSCT (9 ALL, 6 AML) [48]. Haplo apheresis products (CD3^+^ depleted/CD56^+^ selected) were immediately infused (median 18.6 × 10^6^/kg) into pediatric patients conditioned with clofarabine, etoposide, and cyclophosphamide. The treatment regimen included *in vivo* IL-2 following the adoptive transfer. No GvHD, cytokine storm, or infusion syndrome was noted, and donor chimerism peaked around 7–14 days with 100% donor achieved in 16 patients (though 2 had 0% donor). Of the patients who had not undergone HSCT, 7 achieved CR or CR with incomplete count recovery (CRi), 3 partial response (PR), and 4 no response (NR), with 12 proceeding to HSCT; currently, 36% are alive with no evidence of disease (NED). Of those who relapsed following HSCT, 7 achieved CR/CRi and 3 PR with 27% alive with NED. Following these successful studies, the results from the St. Jude AML08 study are eagerly anticipated. In this trial, standard-risk AML patients and high-risk patients without suitable HSCT donors (or who decline HSCT) were eligible for KIR-mismatched haplo NK cell adoptive immunotherapy.

A phase II study investigated the feasibility and safety of haplo NK adoptive transfer for relapsed or persistent myeloid malignancy after allogeneic HSCT and included both pediatric and adult patients [49]. Six patients with AML and 2 with MDS (4 pediatric and 4 adult) were enrolled; 7 had previously received a matched-related donor (MRD) HSCT, and 1 received a matched-unrelated donor (MUD) HSCT. Cyclophosphamide was used for lymphodepletion in 5 patients prior to NK transfer. A median of 10.6 × 10^6^/kg haplo NK cells (CD3^+^ depleted/CD56^+^ selected) was infused (with a median of 2.1 × 10^3^/kg CD3^+^, max 4 × 10^4^/kg), followed by *in vivo* IL-2. No infusion reactions and no GvHD were noted. Interestingly, one patient with mild skin GvHD prior to NK adoptive transfer had resolution of disease. Two nonhematologic AE were observed (both of which resolved): grade 4 respiratory tract infection requiring mechanical ventilation and grade 2 *Clostridium difficile* colitis. Three responses were noted including 2 patients who achieved CR, though both relapsed (survival of 20 months post-NK transfer compared to 5.4 months in patients without a response). In addition, a patient with MDS had resolution of dysplastic features but persistent clonal karyotype abnormality. Studies of NK cell chimerism did not detect any circulating haplo NK cells after infusion. In addition, there was no association between specific NK phenotype, effector function, KIR mismatching, or circulating CD56^+^ cell number postinfusion and response.

### 3.2. Adult Studies

Soon after the first pediatric studies were published, the group at University of Minnesota published a similar study in adults which included 43 patients (24 patients with solid malignancies and 19 with AML—primary refractory, relapsed not in CR without HSCT, or relapsed at least 3 months post-HSCT) [50]. Apheresis was performed on haplo donors, and products underwent CD3^+^ depletion without CD56^+^ selection (resulting in 40% NK cells), which were then cultured overnight with IL-2. Following a dose-finding phase on patients with solid malignancies, AML patients received 20 × 10^6^/kg total nucleated cells (TNC), with a final NK dose of 8.5 × 10^6^/kg (CD3^+^ dose 1.75 × 10^5^/kg). TNC also included 25% monocytes and 20% B lymphocytes. AML patients received this infusion after conditioning with cyclophosphamide and fludarabine (leading to pancytopenia and increased endogenous IL-15), followed by exogenous *in vivo* IL-2. AE included one grade 3 event with worsening of preexisting pulmonary effusions and one grade 2 event with mild hypoxia requiring oxygen support (as well as a biopsy-proven drug rash). Importantly, one patient (post-UCB transplantation) had 100% engraftment of haplo NK cells but died of EBV-related posttransplant lymphoproliferative disorder (PTLD) in the setting of a large number of circulating CD19^+^ cells of haplo donor (but not UCB or recipient) origin. Engraftment of haploidentical cells was assessed in 15 patients with 8 having at least 1% engraftment; in one patient, individual immune cell populations were evaluated and engraftment of only haplo NK cells was observed. Expansion peaked at day 7. Five patients achieved a morphologic CR, including 75% of those with KIR mismatch versus 13% of those without. Furthermore, circulating NK cells represented 51% of lymphocytes in those achieving CR versus 8% in those without CR.

Another study investigated the use of donor lymphocyte infusion enriched for NK using CD56^+^ positive selection [51]. The first infusion was given 6–8 weeks following allogeneic HSCT with nonmyeloablative conditioning (fludarabine-based with alemtuzumab) in 30 adult patients with hematologic malignancies (14 undergoing MRD HSCT and 16 undergoing mismatched-related donor (MMRD) HSCT). High-risk patients were eligible to receive up to 2 additional infusions at 8-week intervals. With cell doses limited to cap CD3^+^CD56^−^ doses (1 × 10^6^/kg for MRD setting and 0.5 × 10^6^/kg for MMRD setting), median CD56^+^CD3^−^ NK doses were 10.6 × 10^6^/kg for MRD setting (median CD3^+^CD56^−^ 0.53 × 10^6^/kg) and 9.21 × 10^6^/kg for MMRD setting (median CD3^+^CD56^−^ 0.27 × 10^6^/kg). For the MRD setting, 6 patients developed acute GvHD (only 1 grade III-IV). In MMRD patients, 9 developed acute GvHD (only 1 grade III-IV), as well as 1 severe chronic GvHD. Other toxicities were tolerable in the posttransplantation setting. In the MRD setting, estimated 1 year overall survival (OS) was 43% (median follow-up of 12 months), while in the MMRD setting, estimated 1 year OS was 42% (median follow-up of 27 months). NK functional assays demonstrated that of 7 patients with poor NK activity just prior to first infusion, NK function was improved 6–8 weeks later in 4 patients (although interpretation of these assays is limited by the use of whole peripheral blood mononuclear cell counts as opposed to NK cell counts to establish effector : target ratios). Interestingly, no differences in toxicities or survival endpoints were noted based on KIR mismatching or donor activation of KIR.

A later study evaluated haplo inhibitory KIR-mismatched NK adoptive transfer in elderly high-risk AML patients not eligible for allogeneic HSCT [52]. Thirteen patients (age 53–73 years) were enrolled, including 5 with active disease, 2 in molecular relapse, and 6 in morphologic CR. Apheresis products were CD3^+^ depleted/CD56^+^ selected, and patients conditioned with cyclophosphamide and fludarabine received a median of 2.74 × 10^6^/kg NK cells (with <1 × 10^5^/kg T cells), followed by *in vivo* IL-2. No GvHD was noted, though few infections and some IL-2 injection site reactions occurred. A homeostatic increase in endogenous IL-15 was noted, peaking at day 3, with peak donor NK at day 10. Interestingly, in one patient, subsequent NK infusions had a shorter duration of persistence; less increase in endogenous IL-15 was also noted with subsequent infusions. Of the 7 patients with detectable disease, 3 achieved CR lasting 4–9 months. Of the 6 in CR, 3 remained disease-free at 18–34 months of follow-up. This study importantly highlights the safety and feasibility of this approach in older adults who are ineligible for HSCT.

Two studies out of Korea also investigated the feasibility and safety of NK adoptive transfer. In the first, 14 patients (11 AML, 1 ALL, and 2 MDS) receiving MMRD T-replete HSCT with reduced intensity conditioning (RIC) received an NK infusion between day +43 and +50 [53]. NK cells were generated from CD34^+^ cells (obtained from an extra day of donor apheresis) cultured *in vitro* with stem cell factor, Flt-3 ligand, IL-7, and hydrocortisone for 3 weeks followed by NK maturation with IL-15, IL-21, and hydrocortisone for an additional 3-4 weeks. Three products were unused due to expansion failure/culture contamination or CD3^+^ count > 10%. Patients received a median of 9.28 × 10^6^/kg cells, with a median of 64% CD122^+^CD56^+^ and 1% CD3^+^ (0–2.6%). Compared to fresh cells, cultured cells had lower expression of inhibitory KIRs and higher expression of activating KIRs and did not require prior IL-2 exposure for *in vitro* cytotoxicity. The donor/recipient pairs included 4 with KIR mismatch, 8 without, and 2 unknowns. No acute toxicities were noted, and 5 cases of GvHD were seen (1 grade II acute and 4 chronic: 2 mild, 1 moderate, 1 severe). Of the 6 leukemia patients who were in CR (1 in CR1 and 5 in CR2+) at the time of HSCT, 4 relapsed (3 deaths), 1 had sudden unexplained death, and 1 was alive with NED. Of the 6 patients with refractory disease, 4 achieved CR (though 3 relapsed and 1 remains alive with NED; median follow-up 18.7 months). The success of NK cell generation in this study led to a subsequent study with some modifications, including the use of very high-dose NK cell adoptive immunotherapy generated through CD3^+^ depletion followed by *in vitro* expansion with IL-15, IL-21, and hydrocortisone [54]. Products were >90% CD56^+^CD122^+^, and 67% exhibited a mature phenotype (CD56^+^CD16^+^). 41 adult leukemia patients (mostly AML with refractory disease) underwent haplo HSCT with RIC (18% had KIR incompatibility). Patients received two NK cell infusions (days +14 and +21) with 0.2 × 10^8^/kg to 2 × 10^8^/kg per infusion (CD3^+^CD56^−^ < 0.3%, with one exception). No change in circulating NK quantity was noted after adoptive transfer, but more NK with activating receptors were detected. Time to engraftment was not significantly different from historical controls, though 2 graft failures were noted. Acute GvHD was observed in 22% of patients (17% grades II–IV) and chronic in 24% (15% severe). TRM occurred in 27%. These rates were not significantly different from a defined historical control group which included patients undergoing haplo HSCT with RIC either without NK adoptive transfer or with low-dose NK adoptive transfer (0.3–24.5 × 10^6^/kg). However, significantly less disease progression was noted in the high-dose NK setting (46%) compared to historical controls (75%), though other parameters were similar, including CR rate, event-free survival (EFS), and OS. The importance of this study is the determination that patients can tolerate very high doses of NK cells, provided CD3^+^ count remains within reason. Although the historic control group was confounded by inclusion of patients who did not receive NK cells or who received “low” dose NK cells, the study found significantly less disease progression in the high-dose NK patients compared to these controls, though EFS and OS were similar.

Another phase I trial examined the adoptive transfer of haplo NK prior to allogeneic transplantation for 21 patients with myeloid malignancies (8 high-risk AML, 6 IR/HR MDS, and 7 CML) [55]. This study divided patients into 2 cohorts: a phase I dose escalation (15 patients with KIR mismatch) and a phase II expansion (6 CML patients, KIR mismatch not required). Nonmobilized haplo NK donors underwent apheresis and products were CD3^+^ depleted (most did not undergo CD56^+^ positive selection in order to increase yield). Because the maximum-tolerated dose was not found in the dose escalation phase, the phase II dosing was set at 5 × 10^6^/kg CD56^+^ cells (median CD3^+^ 0 × 10^5^/kg, max 1.7 × 10^5^/kg). Patients received peripheral blood-derived T-replete HSCT from MRD (*n* = 13) or MUD (*n* = 8); conditioning included busulfan/fludarabine on days −13 to −10 and NK infusion was given on day −8, followed by *in vivo* IL-2 (daily for 5 days), with thymoglobulin on days −3 to −1. The authors note that thymoglobulin was given to prevent NK from hindering engraftment and to reduce GvHD but acknowledge that this also limited the duration of NK efficacy and propose to eliminate this agent in future studies. NK infusions were fairly well tolerated with no grade 3 AE. Time to engraftment was as expected and acute GvHD occurred in 7 patients (2 grade III, no grade IV) with 6 cases of chronic GvHD (5 extensive). Eleven of the 14 patients with active disease achieved CR, and a strong association was noted between relapse-free survival and higher NK dose (>3 × 10^6^/kg versus <3 × 10^6^/kg) as well as with development of GvHD. Of the entire cohort, 52% of patients died of relapse, and there was a day 100 transplant-related mortality rate of 9%. There was a nonsignificant trend towards improved survival in KIR-mismatched setting (but analysis was limited by the small number of KIR-matched donor/patient combinations).

A very well-executed study from Washington University in St. Louis elicited a cytokine-induced memory-like phenotype in NK cells prior to adoptive transfer into AML patients not undergoing transplantation [56]. Initial *in vitro* studies of the memory-like NK cells (generated through preactivation with IL-12, IL-15, and IL-18 for 12–16 hours followed by 1 week of rest with low-dose IL-15 to allow for differentiation) revealed that memory-like NK cells have very distinct phenotypes on mass cytometry analysis, though KIR expression remained unaltered. These cells demonstrated enhanced IFN*γ* production and cytotoxicity against leukemia cell lines or primary human AML compared to control NK and which in memory-like NK occurred regardless of KIR receptor/ligand mismatching. These memory-like NK cells also demonstrated superior reduction of AML burden and improved OS in a xenograft model compared to control NK cells. In a human trial, patients with relapsed or refractory AML who were not candidates for HSCT underwent fludarabine/cyclophosphamide conditioning and infusion of haplo memory-like NK cells (obtained from apheresis product which underwent CD3^+^ depletion, CD56^+^ selection, and preactivation with IL-12, IL-15, and IL-18 for 12–16 hours), followed by *in vivo* IL-2. The NK cells proliferated and expanded, peaking at 7–14 days (with donor memory-like NK representing over 90% of NK in the peripheral blood at day 7, an average increase of 419-fold). Upon ex vivo restimulation, donor memory-like NK cells from both peripheral blood and bone marrow exhibited robust responses against K562 leukemia targets (significantly increased compared to recipient NK cells). Clinical responses were noted in 5 of 9 evaluable patients, with an overall response rate of 55% and a CR/CRi rate of 45%. No correlation was noted with KIR mismatch or frequency of donor NK in the peripheral blood or bone marrow.

Finally, one study investigated the novel approach of using “off-the-shelf” product-derived NK cells for adoptive transfer [57]. In this study, 7 older adult patients with relapsed or refractory AML and no history of HSCT were treated with a total of 20 NK infusions. Cells were obtained through the culture of an aliquot of 20 × 10^6^ cryopreserved cells in media supplemented with IL-2 until desired cell count was reached (median 24 days). A course of therapy included two infusions of equal cell dose 24 hours apart (two doses were investigated: 1 × 10^9^/m^2^ and 3 × 10^9^/m^2^). Per FDA requirements, cells were irradiated to 1000 cGy prior to infusion, precluding *in vivo* expansion but not limiting cytotoxicity. Patients with stable disease or a reduction in blasts were eligible for additional courses at 3-week intervals. No grade 3-4 toxicities related to the infusion were observed. No significant changes in the percent or absolute number of NK subsets were observed, as well as no significant increase in the cytotoxicity of circulating NK cells. However, the higher dose of NK cells (but not the lower dose) led to a significant increase in IL-1R*α* and IL-6 levels at day 7 and yet an increase in IL-10, IP-10, and VEGF on day 21, suggesting an initial promotion of, but later suppression of, an inflammatory response. No CR was observed, though the blast percent did decrease from 70% to 48% in one patient. While the ability to use “off-the-shelf” NK cell therapy is very attractive and this study demonstrated the safety of this approach, the particular method used in this study is limited by the need for prolonged cell culture. In addition, the requirement for irradiation of infusion products likely significantly limited the potential for efficacy of this therapy.

## 4. NK Cells: Future Implications

Considering the promising data so far obtained about the role of this cell population in the treatment of high-risk leukemia, many groups are investigating new immunotherapy approaches aimed at maximizing NK cell efficacy (Figures 1(c)–1(e)). A human anti-KIR mAb (lirilumab) was generated to bind to all KIR2D inhibitory receptors specific for group 1 and 2 HLA-C alleles and block inhibitor signal transduction [58] (Figure 1(c)). *In vitro* and murine model studies showed that lirilumab efficiently promoted NK cell alloreactivity and killing of otherwise resistant HLA-C group 1+ or group 2+ targets, such as normal and tumor cells [58, 59]. Phase I clinical trials demonstrated that the anti-inhibitory KIR mAb is safe [60], and phase II clinical trials with lirilumab are ongoing. Another approach has been to generate and explore the role of an antihuman NKG2A antibody. Ruggeri L et al. found that immunodeficient mice coinfused with human primary leukemia or Epstein-Barr virus cell lines and NKG2A+ natural killer cells pretreated with antihuman NKG2A were rescued from disease progression [61]. In addition, human NKG2A+ natural killer cells reconstituted in immunodeficient mice from human CD34+ cells were able to kill engrafted human primary leukemia or Epstein-Barr virus cell lines by lysis after intraperitoneal administration of antihuman NKG2A. This anti-NKG2A may promote the antileukemic effect of NKG2A+ NK cells recovering after HSCT or through adoptive infusion of NK cells from MRD or MMRD after chemotherapy, without the need to search for a NK cell alloreactive donor.

The effectiveness of NK cell infusions to induce leukemic remission is limited by lack of both antigen specificity and *in vivo* expansion. To address this issue, an innovative immunoglobulin-based strategy is the use of bispecific or trispecific antibodies, known as bispecific or trispecific killer engagers (BiKE, TriKE) (Figure 1(d)). Gleason et al. showed that bscFv CD16/CD19 and tscFv CD16/CD19/CD22 engagers directly bind NK cells and target cells and trigger NK cell activation through CD16, significantly increasing NK cell cytolytic activity and cytokine production against various cell lines [62]. Moreover, Wiernik et al. previously generated a BiKE containing scFv against CD16 and CD33 to create an immunologic synapse between NK cells and CD33+ myeloid targets [63]. Subsequently, a novel modified human IL-15 cross-linker was incorporated, producing a 16-15-33 TriKE to induce expansion, priming, and survival, which they hypothesize will enhance clinical efficacy. When compared with the 16-33 BiKE, the 16-15-33 TriKE induced superior NK cell cytotoxicity, degranulation, and cytokine production against CD33+ HL-60 targets and increased NK survival and proliferation. Specificity was shown by the ability of a 16-15-EpCAM TriKE to kill CD33^−^ EpCAM^+^ targets. Using NK cells from patients soon after allogeneic stem cell transplantation when NK cell function is defective, the 16-15-33 TriKE restored potent NK function against primary AML targets and induced specific NK cell proliferation *in vitro*. These results were confirmed in an immunodeficient mouse HL-60-Luc tumor model where the 16-15-33 TriKE exhibited superior antitumor activity and induced *in vivo* persistence and survival of human NK cells for at least 3 weeks [64].

Finally, the recent success of chimeric antigen receptor (CAR)-engineered T cells in the treatment of B cell malignancies encouraged several groups in investigating genetic modification of NK cells with CAR constructs (Figure 1(e)). CAR-NK cells can bridge over inhibitory signals displayed by tumor cells activating the innate cytolytic functions as well as the release of proinflammatory cytokines [65]. However, the development of CAR-engineered NK cells for adoptive cancer immunotherapy is still in its embryonic stages. The complexity of manufacturing a reasonable large number of peripheral blood-derived NK cells, the lower gene transfer efficiency in comparison with T cells and the limited *in vivo* expansion and persistence once infused in recipients [66, 67] are the major obstacles for the clinical development of CAR-NK cells. In the attempt to overcome at least one of these hurdles, Shimasaki and colleagues invested hard efforts in improving ex vivo expansion of NK cells to allow multiple infusions [68].

## 5. Summary

NK cells play a key role in the immune response against cancer and are particularly important in haplo HSCT as a treatment of high-risk leukemia in both adult and pediatric patients. Their antileukemic effect is mostly related to the presence of “alloreactive” NK cells, though an important role is also played by certain activating KIR (primarily, but not only, KIR2DS1) upon interaction with their HLA class I ligand (C2 alleles). The novel haplo HSCT method recently developed (depletion of *αβ* T and CD19^+^ B cells) allows for the infusion, together with high doses of donor CD34^+^ HSCs, of important effector cells including mature NK cells and *γδ* T cells. Altogether, these recent insights lead to a better control of leukemia recurrence after haplo HSCT.

The abundance of small pilot trials investigating NK adoptive transfer has confirmed that, despite a few instances of infection in the setting of lymphodepleting chemotherapy, this approach is overall feasible and safe, particularly with highly pure infusions of NK cells (to avoid the risk of donor B cell-mediated EBV+ PTLD as likely occurred in one patient) [50]. However, efficacy has yet to be established, due at least in part to the small sample sizes in all of these studies; indeed, although two of the studies reviewed were considered phase II trials, the number of patients enrolled was still extremely small. In the majority of these studies, however, survival rates did not appear to be substantially different than outcomes without NK adoptive therapy. In particular, in the Stern et al. study, NK infusions had no apparent effect on rates of graft failure or relapse as compared to historical controls [46]. While these results are concerning for the future success of NK adoptive immunotherapy, higher numbers of enrolled patients are required to adequately establish the efficacy (or lack thereof) of this approach. Thus, there is an urgent need for the field to now move into larger, randomized trials. In addition, many questions remain unanswered. These include the best timing of NK adoptive transfer in the broad scope of leukemia therapy, ideal disease targets and disease states, preparative regimens for adoptive transfer if not in the immediate post-HSCT setting, and the best methods to enhance NK number (i.e., using cultured cells versus fresh) and NK activity (i.e., which cytokine exposure is ideal and whether the exposure should be *in vitro* or *in vivo*).

These studies describing NK cell development and function, particularly in haplo HSCT, have led to the design of new NK-based immunotherapies to control leukemia relapses, including the employment of a fully human anti-KIR mAb, the use of bispecific/trispecific mAbs linking NK cells to target antigens and/or cytokines, and the generation of NK cells engineered to express a chimeric antigen receptor (CAR) specific for surface tumor antigens. These novel approaches have the capacity to be applied in both HSCT and NK adoptive transfer settings and have the potential to revolutionize the field.
